# High-Resolution Nanoscale AC Quantum Sensing in CMOS Compatible SiC

**DOI:** 10.1021/acs.nanolett.5c02515

**Published:** 2025-07-22

**Authors:** Paul Fisher, Alexander Zappacosta, Jens Fuhrmann, Benjamin Haylock, Weibo Gao, Roland Nagy, Fedor Jelezko, Robert Cernansky

**Affiliations:** † Institute for Quantum Optics, 9189 Ulm University, Albert-Einstein-Allee 11, D-89081 Ulm, Germany; ‡ School of Electrical & Electronic Engineering, 54761 Nanyang Technological University, 50 Nanyang Avenue, Singapore 639798, Singapore; § Institute of Applied Quantum Technologies, Friedrich-Alexander-Universität Erlangen-Nürnberg, 91052 Erlangen, Germany

**Keywords:** quantum sensing, silicon carbide, silicon vacancy, single defect, Synchronized Readout, magnetic field sensitivity, CMOS compatible

## Abstract

High-resolution nanoscale nuclear magnetic resonance (NMR) allows measurement of chemical structure at the single-molecule level for determining molecular dynamics. Until now, nitrogen vacancy centers in diamond have been the only platform to demonstrate single-defect NMR sensing at sub-Hz spectral resolution. Using a single silicon vacancy defect prepared under CMOS-compatible conditions in commercial 4H-silicon carbide at room temperature, we use the Synchronized Readout technique to measure a test signal. We achieve a spectral resolution of 0.33 Hz, necessary for understanding molecular structure, and estimate a magnetic sensitivity of 358 μT/ $\sqrt{Hz}$ for our system. We also explore the necessary improvements for achieving single-proton spin sensitivity. Combining these results with future integrated photonics shows a promising path toward scalable nanoscale sensing for low-cost NMR spectrometers based on an industry-mature silicon carbide material.

## Graphical abstract

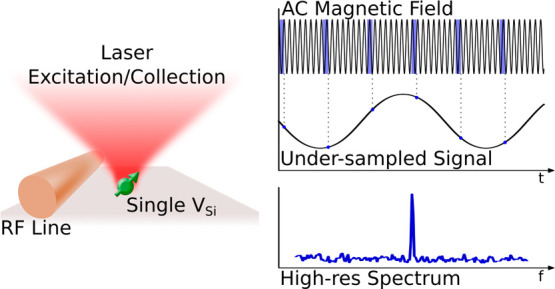

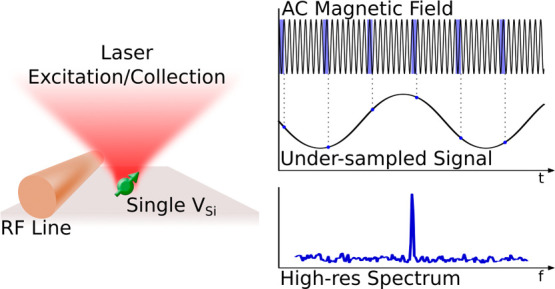

Solid-state spin systems are promising platforms for a variety of quantum applications including communication, computation, and sensing. In many solid-state spin systems such as diamond,^1^ silicon carbide,^2^ or hexagonal boron nitride,^3^ the quantum state of the spin of interest can be optically initialized and detected via a spin-dependent intersystem crossing. To date, diamond is the dominant material platform for nanoscale quantum sensing. It has been used to demonstrate detection of individual proteins,^4,5^ which has enabled study of their molecular dynamics.^6^ Further demonstrations include high-resolution NMR spectroscopy of biological samples at nanoscale by utilizing the quantum heterodyne technique,^7^ and at micron scale using the Synchronized Readout technique.^8^ These powerful approaches extend the spectral resolution of quantum sensors beyond the spin coherence limit of the physical system. CMOS technology has driven the microelectronics revolution of the past several decades, enabling the production of electronic devices at scale and with low enough cost that they have become ubiquitous in our daily lives. Focusing on CMOS compatible materials, with a goal of utilizing existing CMOS industry infrastructure, can provide a pathway to developing on-chip quantum sensors at large scale and ultra low cost. This could lead to a new enabling technology that makes NMR testing widely accessible to a large audience of biologists, chemists and medical scientists. Combining these sensors with integrated photonics would enable large scale NMR testing with spatially separated sensors in parallel, with improved sensitivity and relaxed spatial limitations compared to a wide-field optical setup. An ideal material that can realize these advantages is SiC.

SiC has emerged as a promising material for both quantum and classical technologies. It provides a means to perform full wafer scale fabrication of integrated photonics^9–11^ that are compatible with industrial requirements. The material also hosts many optically addressable spin defects including the silicon vacancy (V_Si_),^12^ divancancy,^13^ and nitrogen vacancy (SiC NV),^14^ which have shown great promise for quantum technologies. While high resolution AC sensing using Synchronized Readout has been shown at the microscale with an ensemble of SiC NV centers,^15^ there has yet to be a demonstration at nanoscale using a single defect. This approach is important for probing single-molecule structure and dynamics at room temperature, to reveal properties inaccessible at the ensemble scale.

V_Si_ defects are very attractive candidates because of their room temperature operation, long electronic coherence times,^13^ spectral stability,^11,16,17^ and ease of integration with photonic structures.^18^ We report a novel preparation of V_Si_ defects under CMOS compatible conditions using nitrogen implantation, resulting in the preparation of single defects implanted within 6.4 nm of the surface with a Hahn echo coherence time of 1.8 μs. These properties are excellent for high-resolution nanoscale NMR sensing due to the close proximity of the defect to surface samples while enabling interactions with AC fields at frequencies above 1 MHz. We perform nanoscale sensing of a test signal oscillating at 3.3 MHz, using the Synchronized Readout technique to achieve a spectral resolution of 0.33 Hz. Measuring with sub-Hz resolution is necessary for measuring molecular J-couplings, which is important for understanding the chemical composition of a sample.^8^ Furthermore, we estimate the magnetic sensitivity of our sensor to be 358  μT/ $\sqrt{Hz}$ , limited by photon shot noise. Finally, by introducing the single defect in a waveguide to enhance collection efficiency,^18^ and with a 87-fold extension to the coherence time, our theoretical model predicts the ability to detect a single protein. These results show great promise for V_Si_ defects to be excellent candidates for nanoscale sensing.

For our measurements we probe the k-site V_Si_ (V_2_),^12^ created in our test sample (see Supporting Information), which has demonstrated microwave addressable spin dynamics at room temperature.^19^ Figure 1a illustrates the energy level structure of the V_2_ in an external magnetic field. The ground state manifold is described by the Hamiltonian,

1 $$H=D\left[S_{z}^{2}-S\left(S+1\right)/3\right]+E\left(S_{x}^{2}-S_{y}^{2}\right)+g\mu_{B}B_{0}S$$

with Landé g-factor *g*, Bohr magneton μ_*B*_, zero-field splitting parameters *D* and *E*, external magnetic field *B*_0_ aligned with the *z*-axis, and spin *S* = 3/2. The ground state spin initialization and readout can be performed optically through an intersystem crossing mechanism in the shelving state.^20^ The spin states *S* = | ± 1/2| decay more frequently from the excited state to the shelving state than the states *S* = | ± 3/2|, providing an optical contrast between the states of up to 14% at cryogenic temperatures,^21^ and up to 6% in shallow defects at room temperature.^22^ Additionally, the shelving state decays preferentially to the *S* = | ± 1/2| states, providing a mechanism for optical initialization.

**Figure 1:**
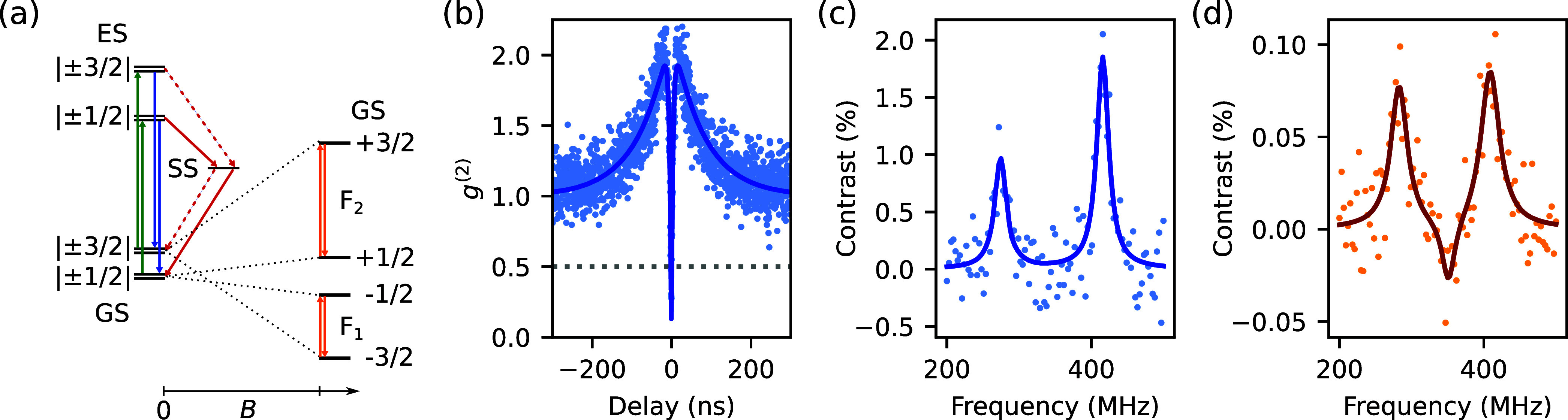
(a) Left: Energy level structure of V_Si_. At zero magnetic field, the ± 1/2 and ± 3/2 spin ground states (GS) are separated by 70 MHz. Illumination with a laser at 785 nm transitions the defect off-resonantly into the excited state (ES), where it can emit fluorescence at 916 nm as it relaxes back to the ground state. Red solid arrows indicate the more likely transitions when the defect relaxes to and from the shelving state (SS). Right: At higher magnetic fields, the ground state manifold becomes split, leading to two peaks in the ODMR spectrum at F_1_ and F_2_, separated by 140 MHz. (b) *g* ^(2)^ autocorrelation measurement of the single defect. Fit reaches a minimum of 0.11(6). (c) and (d) are the ODMR measurements of the single defect and the ensemble, respectively. The external magnetic field strength is estimated to be 12 mT. Asymmetry in height between the peaks is a result of magnetic field misalignment relative to the silicon carbide *c*-axis. The reduction in contrast for the ensemble measurement is due to the presence of non-V_2_ defects that are insensitive to the driving RF field.

We identified a single V_2_ defect first by comparing two photoluminescence maps, each taken with different optical filtering, and then by Optically Detected Magnetic Resonance (ODMR). Since the zero phonon line of V_2_ defects lies at 916 nm, defects that are only present when taking a photoluminescence map filtered between 900 and 1000 nm, and not present when taking taking a map filtered between 850 and 900 nm, are identified as V_2_. We selected such a defect and confirmed its identity using ODMR (Figure 1c). These measurements were taken using a 785 nm diode laser at a power of 1 mW into the microscope objective.This two-step method identified candidate single defects faster than systematic ODMR measurements on isolated defects alone. The peaks at around 270 and 410 MHz correspond to the – 3/2 ↔ – 1/2 and +1/2 ↔ + 3/2 transitions of the V_2_ ground state, respectively, with an external magnetic field strength of ∼ 12 mT.^19^ We also confirmed that it is a single defect with a *g* ^(2)^ autocorrelation measurement (Figure 1b). We measured *g* ^(2)^(0) = 0.11(6), surpassing the photon antibunching threshold of 0.5.

We measured the coherence properties of the single defect, and a dense ensemble for comparison. We used the +1/2 ↔ + 3/2 transition at ∼ 410 MHz for the spin control and quantum sensing measurements, with Rabi and Hahn-Echo decay measurements shown in Figure 2. After a π-pulse, the single defect and the ensemble show maximum peak-to-peak contrasts of 4.552(96) % and 0.346(6) %, respectively. From the Hahn-Echo measurements, we estimate the T_2_ coherence times to be 1831(161) ns and 1049(87) ns for the single defect and the ensemble, respectively. These coherence times are at least 80 times shorter than those observed at room temperature in deeply implanted V_2_ defects^19^ and limit the spectral resolution of the quantum sensor to be greater than 500 kHz. To extend sensing capabilities to arbitrary resolution, we use the Synchronized Readout measurement scheme.^8^

**Figure 2:**
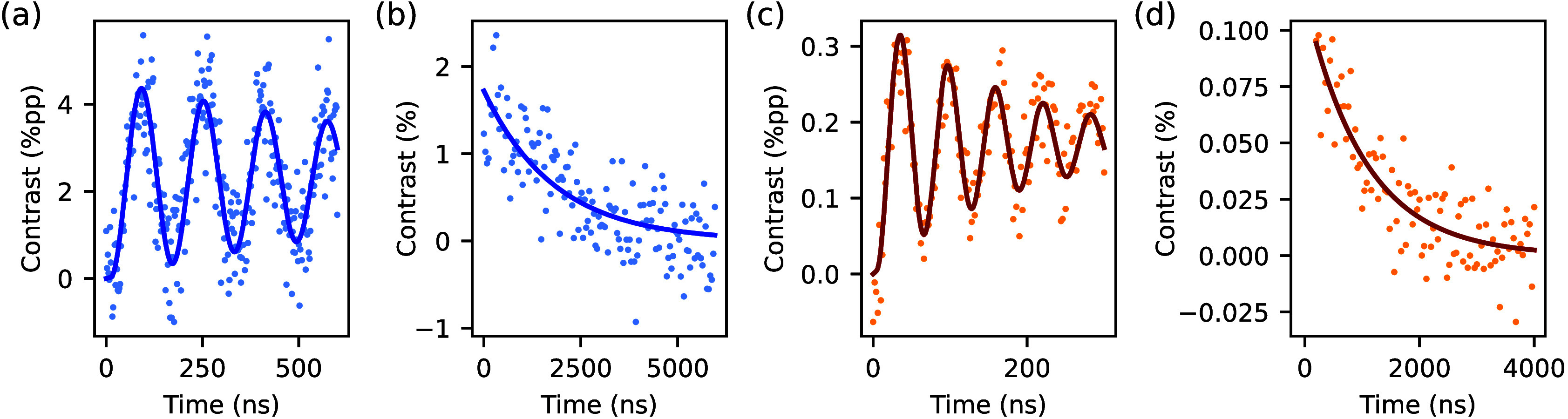
(a) and (b) are the Rabi and Hahn echo measurements for the single defect, respectively. (c) and (d) are the Rabi and Hahn echo measurements for the ensemble, respectively. Exponential fits to the Hahn echo measurements give T_2_ times of 1831(161) ns and 1049(87) ns for the single defect and the ensemble, respectively. Uncertainties are from the confidence bounds of the fits.

Synchronized Readout extends the frequency resolution of AC quantum sensing by taking repeated, regularly spaced samples of an AC magnetic field with reinitialization of the sensor after every sample. This decouples the frequency resolution from the sensor properties, leaving them to determine bandwidth and sensitivity. The ultimate limit is determined by the timing stability and memory capacity of the measurement equipment. Figure 3 demonstrates the measurement protocol schematically. Consider an AC magnetic field that varies with time, *t*, of the form *B* cos­(2*πνt* + ϕ) where *B* is the magnetic field amplitude, ν is the signal frequency, and ϕ is the phase of the AC field. The additional phase accumulated by the defect’s spin state due to the AC field during a Hahn echo measurement is given by,

2 $$\Phi \left(t\right)=\frac{2g\mu_{B}B}{\pi \hbar \nu }\sin^{2}\left(\frac{\pi \nu \tau }{2}\right)\sin\left(2\pi \nu t+\pi \nu \tau +\phi \right)$$

with *ℏ* as the reduced Planck constant and τ as the free precession time (ideally τ = 1/ν) of the Hahn echo sequence. The final π/2-pulse of the Hahn echo sequence rotates the additional phase onto the *z*-axis of the defect’s Bloch sphere where it will be reflected in the expected spin measurement as ⟨*S*⟩ = 1 + 0.5 sin­(Φ­(*t*)).

**Figure 3:**
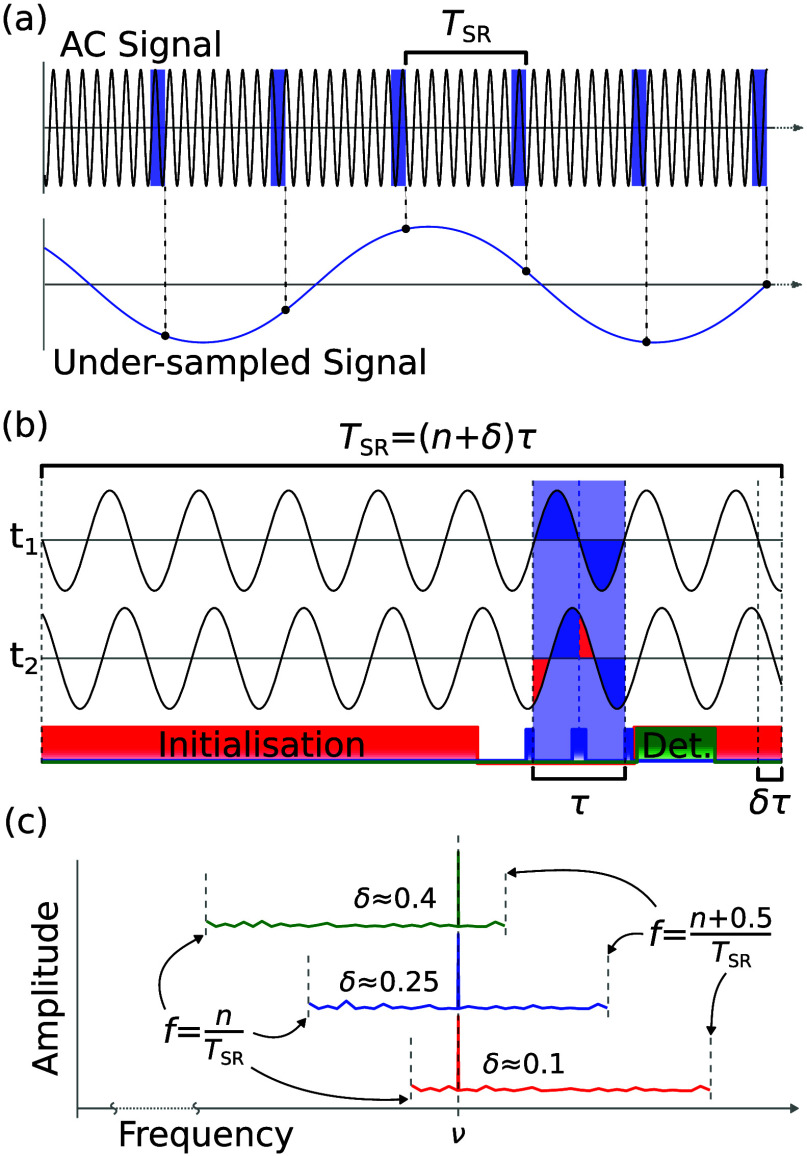
(a) The AC signal to be measured. Highlighted are the repeated Hahn echo measurements spaced by a regular period *T*_SR_. Below is the under-sampled signal recovered by the measurements. (b) Schematic of a single Synchronized Readout pulse sequence unit. The Hahn echo measurement is shown sampling the AC signal at two different times *t*_1_ and *t*_2_. The phase accumulated by the defect spin is different owing to the phase shift caused by the δ component of *T*_SR_. Below is the pulse sequence repeated every period, with laser (red), detection (green), and RF pulse sequences (blue). The RF pulses for the Hahn echo sequence are π/2_*x*_, π_*x*_, and π/2_*y*_. (c) Discrete Fourier transform of the under-sampled signal, using three different values of δ.

Performing measurements with a regular period of *T*_SR_ = (*n* + δ)/ν, for integer *n* ≥ 0 and 0 < δ ≤ 0.5, samples the expected spin over time. The positive discrete Fourier transform of this sequence reveals the frequency spectrum of the signal with a bandwidth of 1/(2*T*_SR_) and resolution given by the inverse of the measurement interval. For *n* ≥ 1, the oscillation of the AC field of interest will be under-sampled and the measured signal is a low frequency alias of the true signal. The true frequency is recovered by adding a frequency correction of *n*/*T*_SR_. The choice of δ determines the location of the frequency ν in the recovered spectrum, with δ = 0.25 placing it in the center. Synchronizing the phase of the AC signal with the measurement interval, either by locking it to the beginning of the spin polarization pulse in NMR, or with a phase trigger in the case of our test signal, enables coherent averaging of successive measurement intervals.

Figure 4a,b shows the frequency-corrected discrete Fourier transform of the Synchronized Readout measurement for our single defect and ensemble, respectively. The artificial signal was set with a period of 300 ns (∼3.3 MHz), to match the free precession time of the Hahn echo sequence. This frequency is suitable for sensing nuclear spins under low magnetic fields (∼100 mT).^8^ The test signal phase was locked with the beginning of the measurement interval. We collected 12000 measurement intervals each of 3 s, resulting in a frequency resolution of ∼ 0.33 Hz and taking 10 h total. The sampling period *T*_SR_ of the signal was 2472 ns, resulting in a measurement bandwidth of 202 265 Hz. *T*_SR_ includes 1000 ns laser time for initialization, 800 ns wait between the laser and Hahn echo sequence to ensure the defect is not in the shelving state, 300 ns for free precession time, and 300 ns optical detection time. The remaining 72 ns is an offset for placing 3.3 MHz in the middle of the Synchronized Readout spectrum (δ ≈ 0.25), with respect to a limitation of our pulse generator that the sampling period must be a multiple of 8 ns. The measured test signal at 0.5 V̇pp has a contrast of 1.142% with a noise level of 0.297%. We performed Synchronized Readout with the same parameters on our spin ensemble and achieved a contrast of 7.17 × 10^–2^ % with a noise level of 2.75 × 10^–2^ %.

**Figure 4:**
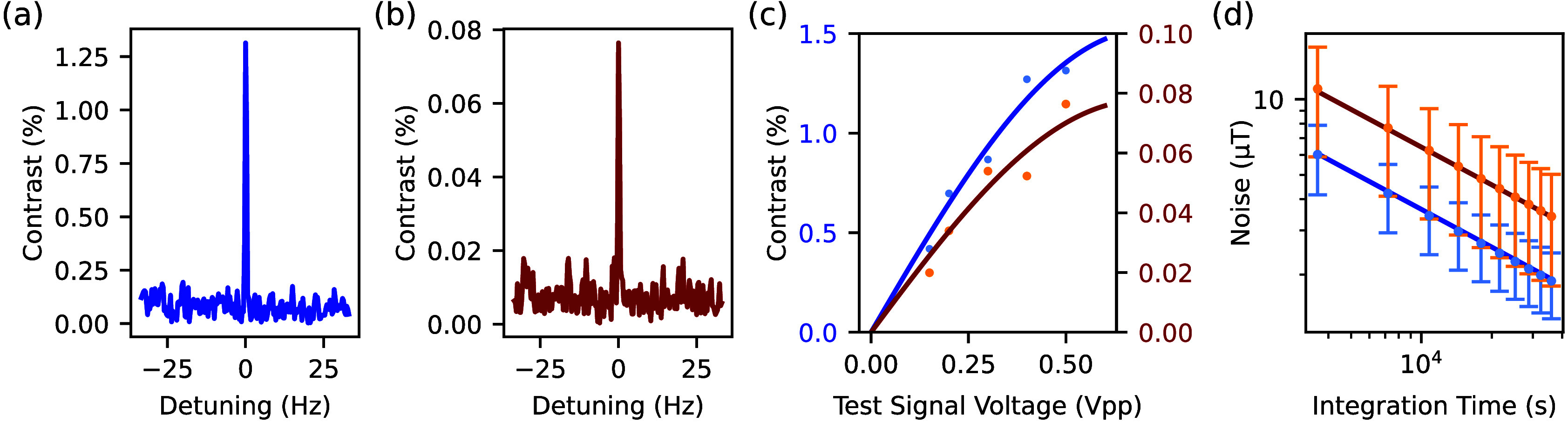
(a) and (b) are the discrete Fourier transforms of the Synchronized Readout measurements on the single defect and the ensemble, respectively, using a test signal amplitude of 0.5 V̇pp and focusing on a 66 Hz window centered on the test signal frequency (3 333 333 Hz). The spectral resolution is ∼ 0.33 Hz. (c) Calibration of the test signal magnetic field strength for the single defect (blue) and the ensemble (orange). The fits estimate maximum contrasts of 1.52(32) % and 0.08(3) %, when the test signal strength is 46.7 μT, and maximum phases per V̇pp of 2.20(63) rad and 2.25(102) rad for the single defect and the ensemble, respectively. Uncertainties are from the confidence bounds of the fits. (d) Change in noise amplitude over time for single defect (blue) and ensemble (orange). The error bars include the standard deviation of all five noise measurements, one for each test signal voltage, plus the peak contrast uncertainty from the test signal magnetic field strength calibration.

Next we estimated the magnetic sensitivity of our single defect sensor. The theoretical sensitivity is given by the formula,^23^

3 $$\eta =\frac{\pi \hbar \sqrt{T_{SR}}}{2g\mu_{B}\tau F}e^{{\left(\frac{\tau }{T_{2}}\right)}^{2}}$$

with $F=1/\sqrt{1+1/\left(C^{2}n_{avg}\right)}$ as the readout fidelity, *C* as the optical spin contrast, and *n*_avg_ as the average number of photons received per single measurement. For our single defect, this gives us a theoretical sensitivity of approximately 154(32) μT/ $\sqrt{Hz}$ . Our contrast (1.52(30) % from Hahn echo measurement) and number of photons per Hahn echo sequence (4.2 × 10^–4^) provides a low readout fidelity of 3.1 × 10^–4^. This results in a noise level 3210 times greater than the single electron spin projection limit of approximately 16.3 nT/ $\sqrt{Hz}$ .

Experimental magnetic sensitivity was estimated using our Synchronized Readout measurements following the method of ref 8. We first calibrated a correspondence between our signal contrast and magnetic field strength using Synchronized Readout measurements at multiple test signal amplitudes (Figure 4c). This calibration tells us that the signal amplitude detected by the single defect is 32.69(936) μT when the measured Synchronized Readout contrast is 1.142%. This also provided a magnetic field strength for the noise level in the Synchronized Readout measurements. Plotting the change in magnetic field noise over the 10 h for all our Synchronized Readout measurements (Figure 4d) shows a $1/\sqrt{t}$ correspondence. From these fits, we find sensitivities of 359(103) μT/ $\sqrt{Hz}$ and 653(289) μT/ $\sqrt{Hz}$ for the single defect and the ensemble, respectively. This result for the single defect is more than two times larger than the theoretical value. We believe this discrepancy can be explained by imperfections in our experimental apparatus, pulse sequences, and other electronic noise.

In the case of the ensemble, there appears to be a more significant source of noise. When applying eq 3 to an ensemble, the sensitivity should improve by a factor of $1/\sqrt{N}$ , where *N* is the number of spins present. The result of eq 3 using the ensemble parameters yields a sensitivity of approximately 309  μT/ $\sqrt{Hz}$ . With the same factor of 2 seen in the single defect sensitivity, this suggests there is only one spin present in the ensemble. This is not a realistic situation and there must be an additional major source of noise. One candidate is state mixing due to misalignment of our external magnetic field. This is indicated by the negative peak present in Figure 1d, and could be an important factor which must be addressed in future experiments.

In comparison with single defect sensing in diamond, our measured sensitivity is much more coarse. Sensitivities as fine as 9.1 nT/ $\sqrt{Hz}$ have been measured using a single NV center in diamond.^24^ While a diamond NV center and a V_Si_ defect may technically share the same spin projection limit to sensitivity, the disadvantage of V_Si_ defects lies in readout fidelity. V_Si_ exhibit lower fluorescence and a low ODMR contrast. Furthermore, the alignment of the V_Si_ dipole axis nearly perpendicular to the surface means that the majority of fluorescence is emitted parallel to the surface and trapped by total internal reflection. Efforts to improve fluorescence emission and collection include quenching techniques during defect creation,^25^ and the creation of solid immersion lens^19^ and waveguide^18^ structures. ODMR contrast can be effectively doubled using two-frequency driving, and has been used to demonstrate a sensitivity of 1.4  μT/ $\sqrt{Hz}$ in an ensemble of V_Si_ defects.^26^ In comparison with other platforms, ensembles in hexagonal boron nitride have demonstrated a sensitivity of 1  μT/ $\sqrt{Hz}$ ,^27^ while an ensemble in a pentacene-doped organic crystal has achieved 405 nT/ $\sqrt{Hz}$ .^28^ An ensemble in diamond has achieved a sensitivity of 210 fT/ $\sqrt{Hz}$ due to improvements to optical collection and noise reduction techniques that enabled sensing with a free precession time of 100 μs.^29^

Finally, we evaluate the possibility of measuring a single protein using our nanoscale quantum sensor. Our primary limitation is the low optical readout fidelity, stated earlier as 3.1 × 10^–4^. Assuming a 1 s integration time, we estimate that we can measure approximately 5.26 × 10^6 1^H nuclear spins situated above the 4H-SiC surface.^5^ To reach single spin sensitivity, we must improve the readout fidelity, increase T_2_ time, and implant the sensor closer to the surface. To improve the fidelity, we can implant our sensor in a waveguide to achieve count rates of 180 000 photons per second, as in ref 18. This reference also achieved T_2_ times up to 42.5 μs. We can also use the best contrast amplitudes of 3% as found in ref 22, and enhance this contrast further to 6% using the two-frequency driving technique described in ref 26. Furthermore, from simulations using SRIM,^30^ we hypothesize that it is possible to prepare defects at a depth of 3 nm using 1 keV of ion energy during implantation. Under these conditions, it is possible to measure approximately 8 ^1^H nuclear spins. To reach single spin sensitivity and beyond, it is necessary to increase T_2_ coherence time to at least 160 μs by using CPMG pulse sequences,^31^ or increasing readout fidelity with quantum logic.^5^ Material methods of improving coherence time include implantation with lighter ion species to reduce lattice damage, and using isotopically purified SiC.^32^ Although this analysis disregards the compounding effect on spin decoherence and charge state instability of using even more shallow defects, surface treatments may be used in order to mitigate this effect, as has been proposed for other platforms.^33,34^

In conclusion, we have demonstrated for the first time high resolution AC quantum sensing using a single defect implanted in commercial 4H-SiC that has been prepared under CMOS compatible conditions. This work demonstrates the viability of creating shallow defects in silicon carbide for room-temperature quantum sensing via ^15^N^+^ ion irradiation. We estimate that this sensor could detect a signal equivalent to 5 260 000 hydrogen spins, and provide a strategy to achieve single-spin sensitivity that will be investigated in future work. Preparing these sensors in an array of waveguides coupled with standard optical fiber provides a path toward scalable, low-cost, high resolution, nanoscale NMR sensing.

## Supplementary Material



## Notes

Materials and defect implantation, experiment hardware, sensitivity calibration (PDF)
