# Supplementary material for: High-Resolution Nanoscale AC Quantum Sensing in CMOS Compatible SiC
Source: Nano Lett. 2025 Jul 22;25(30):11626–31. doi: 10.1021/acs.nanolett.5c02515 (PMC12314907; doi:10.1021/acs.nanolett.5c02515)
Supplement: Supplementary file 1 [file nl5c02515_si_001.pdf]

Supporting Information for "High-resolution nanoscale AC quantum sensing in CMOS compatible SiC"

Paul Fisher,† Alexander Zappacosta,† Jens Fuhrmann,† Benjamin Haylock,† Weibo Gao,‡ Roland Nagy,¶ Fedor Jelezko,† and Robert Cernansky*,†

†*Institute for Quantum Optics, Ulm University, Albert-Einstein-Allee 11, D-89081 Ulm, Germany*

‡*Nanyang Technological University, Physics Department, 21 Nanyang Link, Singapore 637371, Singapore*

¶*Institute of Applied Quantum Technologies (AQuT.), Friedrich-Alexander-Universität Erlangen-Nürnberg, 91052 Erlangen, Germany*

E-mail: robert.cernansky@uni-ulm.de

Materials and Defect Implantation

Our silicon carbide sample was obtained from JXT and is prepared with an n-doped epilayer to a concentration of $5.92 \times 10^{14}\,\mathrm{cm}^{-3}$. Defects were created by irradiation with a $^{15}\mathrm{N}^{+}$ ion beam, perpendicular to the silicon carbide surface. The ion energy was $2.5\,\mathrm{keV}$ and we implanted in multiple spots with fluences between $10^{9}\,\mathrm{cm}^{-2}$ and $10^{13}\,\mathrm{cm}^{-2}$. This source was chosen because the method is well established for implanting shallow nitrogen vacancy defects in diamond, and many facilities are already equipped with this capability. As creation of V_{Si} defects only requires the displacement of silicon atoms from the crystal lattice, any source

may be chosen. The only consideration being the potential for local doping of the crystal lattice with the ion species, which can reduce spin coherence by the presence of additional spins. The sample was then annealed at 450 °C for 1 hour (with 1 h ramp-up and ∼3 h cool-down) under vacuum ($<5 \times 10^{-6}$ Pa). This temperature and duration was chosen as it has been shown to improve fluorescence of V_{Si} defects created by carbon ion implantation,[1] while remaining below the 500 °C threshold for SiC CMOS compatibility. Implantation yield of any optically-active defects, including non-V_2 defects that are insensitive to RF driving, was estimated by counting over four confocal images each in the 10^9 cm^{-2} and 10^{10} cm^{-2} regions and was found to be 1.5(5) %. Modelling using SRIM estimates the mean implantation depth as 6.4 nm, meaning defect creation is in the same depth range. Figure S1a and S1b show confocal images of sections of the 10^{10} cm^{-2} and 10^{13} cm^{-2} regions we measured in this experiment.

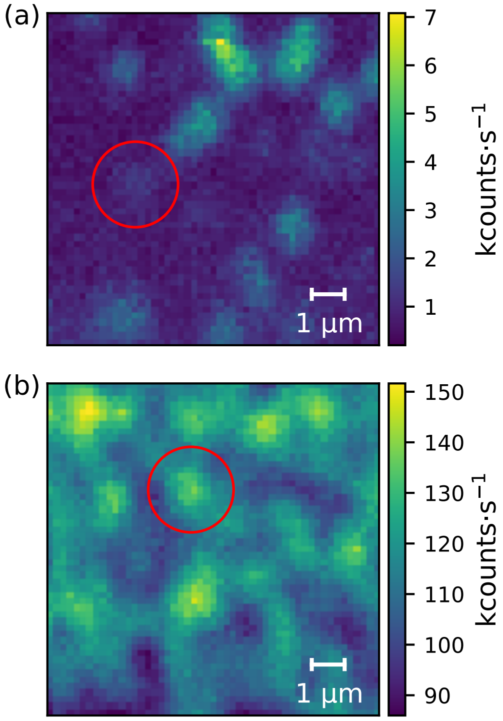

Figure S1: Scanning confocal microscope images of our sample containing the single defect (irradiation dose 10^{10} cm^{-2}) (a) and the ensemble (irradiation dose 10^{13} cm^{-2}) (b). Regions measured in this experiment are circled.

Experiment Hardware

Our measurements were performed using a home-built confocal microscope with a fixed optical path. We used a 785 nm diode laser, for off-resonant excitation of our defects, that was modulated using an acousto-optic modulator (G&H AOMO 3200-124). The confocal microscope had a 0.9 NA air objective (Zeiss EC Epiplan-NEOFLUAR 100x/0.9 DIC) for laser focusing and fluorescence collection. The laser input to the microscope was filtered with an 800 nm shortpass filter to reject laser noise from the colour centre fluorescence (Thorlabs FESH0800). The collection path was filtered using a stack of two 900 nm longpass filters and one 1000 nm shortpass filter (Thorlabs FELH0900, FESH1000). The filters used in creating a photoluminescence map without V_2 defects were two 850 nm longpass filters and one 900 nm shortpass filter (Thorlabs FELH0850, FESH0900). Laser and collection paths were split using an 805 nm dichroic mirror (Thorlabs DMLP805). Collected fluorescence was focused into optical fibre and passed to a fibre-based 50:50 coupler (Thorlabs TW930R5A1) then to a pair of silicon single photon APDs (Excelitas SPCM-AQRH-14-FC-ND). The $g^{(2)}$ autocorrelation data was collected and processed using a coincidence counter (Swabian Instruments Timetagger Ultra), while all other data was collected using a National Instruments DAQ (NI USB-6343). The sample was mounted to a 3-axis piezo stage (Piezoconcept LT3-200) to move the sample into the fixed focus of the microscope. During every experiment, the sample position was refocused by the piezo stage every 5 minutes to counteract stage drift and keep the fluorescence constant.

For our spin control measurements, RF was delivered to the sample using a 20 µm diameter copper wire stretched across the surface of the sample. Our defects were located approximately 5 µm to 10 µm away perpendicular to the edge of the wire. Spin control pulses were created using a vector signal generator (Siglent SSG5060X-V) with external IQ modulation, digitally-controlled switching (Minicircuits ZASW-2-50DRA+), and an RF amplifier (Minicircuits ZHL-5W-1+). The test signal was generated using a function generator (Rigol DG1000Z) and combined with the amplifier output using a frequency diplexer. IQ

control, and optical and RF switching were orchestrated using a Swabian Instruments Pulsestreamer 8-2. The external magnetic field was supplied by a pair of stacked cylindrical samarium-cobalt magnets (1 cm tall × 1 cm diameter each) mounted beneath the sample and movable using a 3-axis stage. Its motion was independent of the sample stage so the magnetic field was fixed relative to the microscope focus.

Sensitivity Calibration

We estimated the magnetic field amplitude of our test signal by rearranging Main Text Eq. 2 with $\nu = 1/\tau$, the sinusoidal component = 1, and for a phase of $\pi/2$. This corresponds to maximum signal amplitude with a magnetic field of 46.7 µT. This value, combined with the fit of signal contrasts for many test signal amplitudes (Main Text Figure 4c), was used to calibrate the magnetic field amplitude of the noise. This follows the function $46.7 \sin^{-1}(A_{\mathrm{meas}}/A_{\mathrm{max}})$ µT, with $A_{\mathrm{meas(max)}}$ being the measured(maximum) amplitude from the fit. The noise was recovered by taking the standard deviation of all points in the Synchronised Readout spectrum, except for 21 points centred on the test frequency peak, and 10 points at the beginning of the spectrum.

References

(1) Wang, J.-F.; Li, Q.; Yan, F.-F.; Liu, H.; Guo, G.-P.; Zhang, W.-P.; Zhou, X.; Guo, L.-P.; Lin, Z.-H.; Cui, J.-M.; Xu, X.-Y.; Xu, J.-S.; Li, C.-F.; Guo, G.-C. On-Demand Generation of Single Silicon Vacancy Defects in Silicon Carbide. *ACS Photonics* **2019**, *6*, 1736–1743.
